# Generation and characteristics of a novel “double-hit” high grade B-cell lymphoma cell line DH-My6 with *MYC*/*IGH* and *BCL6*/*IGH* gene arrangements and potential molecular targeted therapies

**DOI:** 10.18632/oncotarget.26060

**Published:** 2018-09-11

**Authors:** Hiroaki Kikuchi, Tomonori Higuchi, Yumiko Hashida, Ayuko Taniguchi, Mikio Kamioka, Takahiro Taguchi, Akihito Yokoyama, Ichiro Murakami, Mikiya Fujieda, Masanori Daibata

**Affiliations:** ^1^ Department of Microbiology and Infection, Kochi Medical School, Kochi University, Nankoku, Kochi 783-8505, Japan; ^2^ Department of Pediatrics, Kochi Medical School, Kochi University, Nankoku, Kochi 783-8505, Japan; ^3^ Department of Hematology and Respiratory Medicine, Kochi Medical School, Kochi University, Nankoku, Kochi 783-8505, Japan; ^4^ Department of Laboratory Medicine, Kochi Medical School, Kochi University, Nankoku, Kochi 783-8505, Japan; ^5^ Department of Molecular and Cellular Biology, Kochi Medical School, Kochi University, Nankoku, Kochi 783-8505, Japan; ^6^ Department of Pathology, Kochi Medical School, Kochi University, Nankoku, Kochi 783-8505, Japan

**Keywords:** double hit lymphoma, MYC, BCL6, PLK1, HDAC

## Abstract

“Double-hit” lymphoma (DHL) is a high-grade B-cell lymphoma that harbors concurrent *MYC* and *BCL2* or *BCL6* rearrangements. Because cases of *MYC*/*BCL6* DHL are uncommon, most reported conclusions have been based on cases of *MYC*/*BCL2* DHL. Lack of experimental *MYC*/*BCL6* DHL models continues to hinder the pathophysiologic and therapeutic investigations of this disorder. We herein describe a novel *MYC*/*BCL6* DHL cell line, designated DH-My6, carrying both the *MYC*–*IGH* and *BCL6*–*IGH* fusion genes. Interruptions of *MYC* and *BCL6* expressions using short interfering RNAs and chemical inhibitors led to significant attenuation of DH-My6 cell growth. Greater antitumor effects were found when the cells were treated with a combination of MYC and BCL6 inhibitors. Moreover, the PLK1 inhibitor volasertib and the HDAC inhibitor vorinostat synergized strongly when combined with the bromodomain inhibitor JQ1. DH-My6 is a new well-validated *MYC*/*BCL6* DHL cell line that will provide a useful model for studies of the pathogenesis and therapeutics for the less common DHL tumor type. The rationale for approaches targeting both MYC and BCL6, and in combination with PLK1 or HDAC inhibitors for superior suppression of the aggressive *MYC*/*BCL6* DHL warrants further *in vivo* testing in a preclinical model.

## INTRODUCTION

“Double-hit” lymphoma (DHL) represents a subset of B-cell malignancies characterized by the presence of *MYC* (8q24) rearrangement and concurrent *BCL2* (18q21) or *BCL6* (3q27) rearrangements [1]. In recognition of its unique biology and clinical behavior, DHL has been included in the 2016 revision of the World Health Organization (WHO) classification of lymphoid neoplasms as a new category of “high-grade B-cell lymphoma (HGBL) with *MYC* and *BCL2* or *BCL6* rearrangements” [2, 3]. Based on reviews in the literature [1, 4, 5], cases of HGBL with *MYC* and *BCL2* rearrangements (*MYC*/*BCL2* DHL) form the great majority of DHLs (60–85%), whereas cases of HGBL with *MYC* and *BCL6* rearrangements (*MYC*/*BCL6* DHL) are relatively rare (5–8%) and even less common than “triple-hit” lymphoma (THL) that involves *MYC*, *BCL2*, and *BCL6* simultaneously (16%). This is because most of what we know about DHLs is based on cases with *MYC*/*BCL2* DHL, which has an inferior prognosis when treated with regimens for diffuse large B-cell lymphoma (DLBCL) and has a very high recurrence rate with a reported median survival of only 0.2 to 1.5 years [1, 6, 7]. In contrast, there are far fewer data available for *MYC*/*BCL6* DHL. Some studies have suggested that the clinicopathologic features of *MYC*/*BCL6* DHL are distinct from those of *MYC*/*BCL2* DHL [8–11]. Cases of *MYC*/*BCL6* DHL more often involve extranodal sites and have less complex karyotypes [9, 10]. In addition, gene expression profiling of MYC^+^BCL2^–^BCL6^+^ lymphoma cells has shown them to be different from MYC^+^BCL2^+^BCL6^–^ lymphoma cells [11]. Thus, *MYC*/*BCL6* DHL is likely a different disease biologically from *MYC*/*BCL2* DHL and remains an incompletely characterized disease entity.

One of the major limitations in understanding the pathogenesis of *MYC*/*BCL6* DHL is the lack of *in vitro* and *in vivo* models by which unlimited supplies of lymphoma cells with concurrent *MYC* and *BCL6* rearrangements can be studied repeatedly and extensively. So far, there have been various lymphoma cell lines that appear to have both *MYC* and *BCL2* rearrangements [12–14]. Most of these cell lines were reported primarily before sufficient recognition of the clinical importance of DHL and have contributed to the study of lymphomas bearing alterations of both *MYC* and *BCL2*. However, they have not been well authenticated genetically against primary lymphoma cells. In this context, generation of *MYC*/*BCL6* DHL cell lines is a prerequisite for increasing our knowledge of the less common forms of DHL and for the identification of valid therapeutic targets.

Herein, we describe a fully characterized lymphoma cell line harboring simultaneous *MYC* and *BCL6* rearrangements, designated DH-My6, that is proved to be immunophenotypically and genetically consistent with a primary DHL tumor. DH-My6 is a new validated *MYC*/*BCL6* DHL cell line carrying both fusion genes of *MYC* with the immunoglobulin heavy-chain (*IGH*) locus (14q32) and *BCL*6–*IGH*. Using this model, we evaluated the potential of MYC- and BCL6-targeted strategies in combination with agents targeting molecules associated with cell proliferation, such as polo-like kinase 1 (PLK1) and histone deacetylase (HDAC) inhibitors, as possible therapeutic approaches to aggressive *MYC*/*BCL6* DHL.

## RESULTS

### Generation and characteristics of the DH-My6 cell line

**Figure 1:**
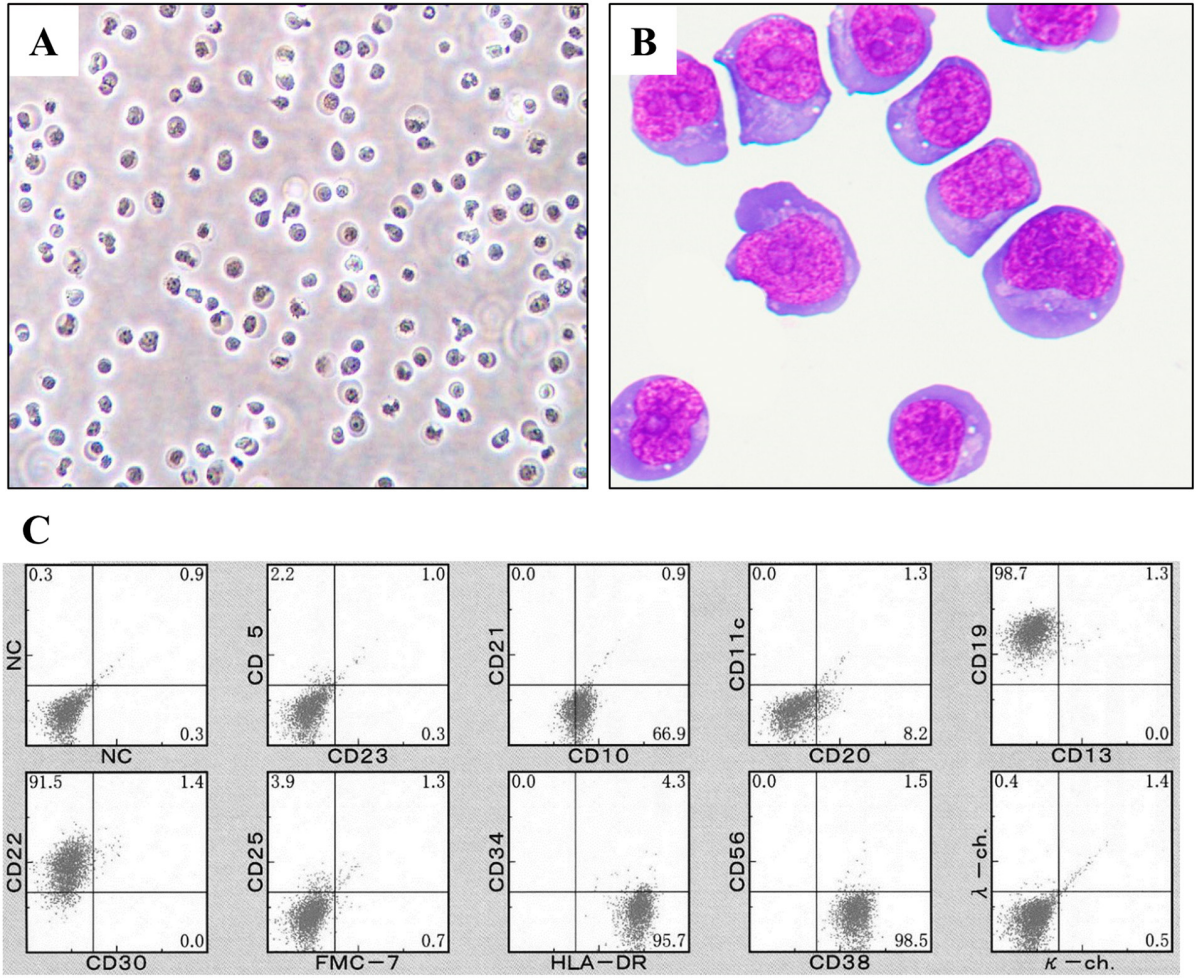
Appearance and surface immunophenotype of DH-My6 cells **(A)** Phase-contrast microphotograph of growing DH-My6 cells. **(B)** Cytospin preparation of DH-My6 cells closely resembling the primary lymphoma cells (May-Giemsa staining). **(C)** Representative flow cytometric histograms of DH-My6 cells.

The DH-My6 cell line was generated from tumor tissue of a patient with *MYC*/*BCL6* DHL. The cells began to proliferate 2 weeks after the initiation of culture and then could be regularly passaged in RPMI 1640 medium supplemented with 10% fetal calf serum (FCS). The cells could be frozen under standard conditions using medium containing 10% FCS and 10% dimethylsulfoxide (DMSO), and could be revived after storage in liquid nitrogen. DH-My6 cells grew in single-cell suspensions with a doubling time of 20 h (Figure 1A). The cell line was composed of medium-to-large-sized cells (Figure 1B). The nuclei were round or slightly irregular with slightly coarse chromatin and had one or more nucleoli. The cytoplasm was basophilic and occasionally contained small vacuoles. The morphology of DH-My6 cells closely resembled the primary lymphoma cells. The cells were shown to be negative for Epstein–Barr virus by polymerase chain reaction (PCR) analysis.

The immunophenotypes of DH-My6 cells were virtually identical to the primary tumor cells. DH-My6 cells were positive for CD10, CD19, and CD22, and negative for CD5, CD11c, CD13, CD21, CD23, CD25, CD30, CD34, CD56, FMC-7, and surface Ig kappa- and lambda-light chains (Figure 1C). The cells had a germinal center B-cell like (GCB) phenotype. Notably, DH-My6 cells express a high level of CD38, and a fraction of weakly CD20-positive or -negative cells was consistently detected during cell passages.

**Figure 2:**
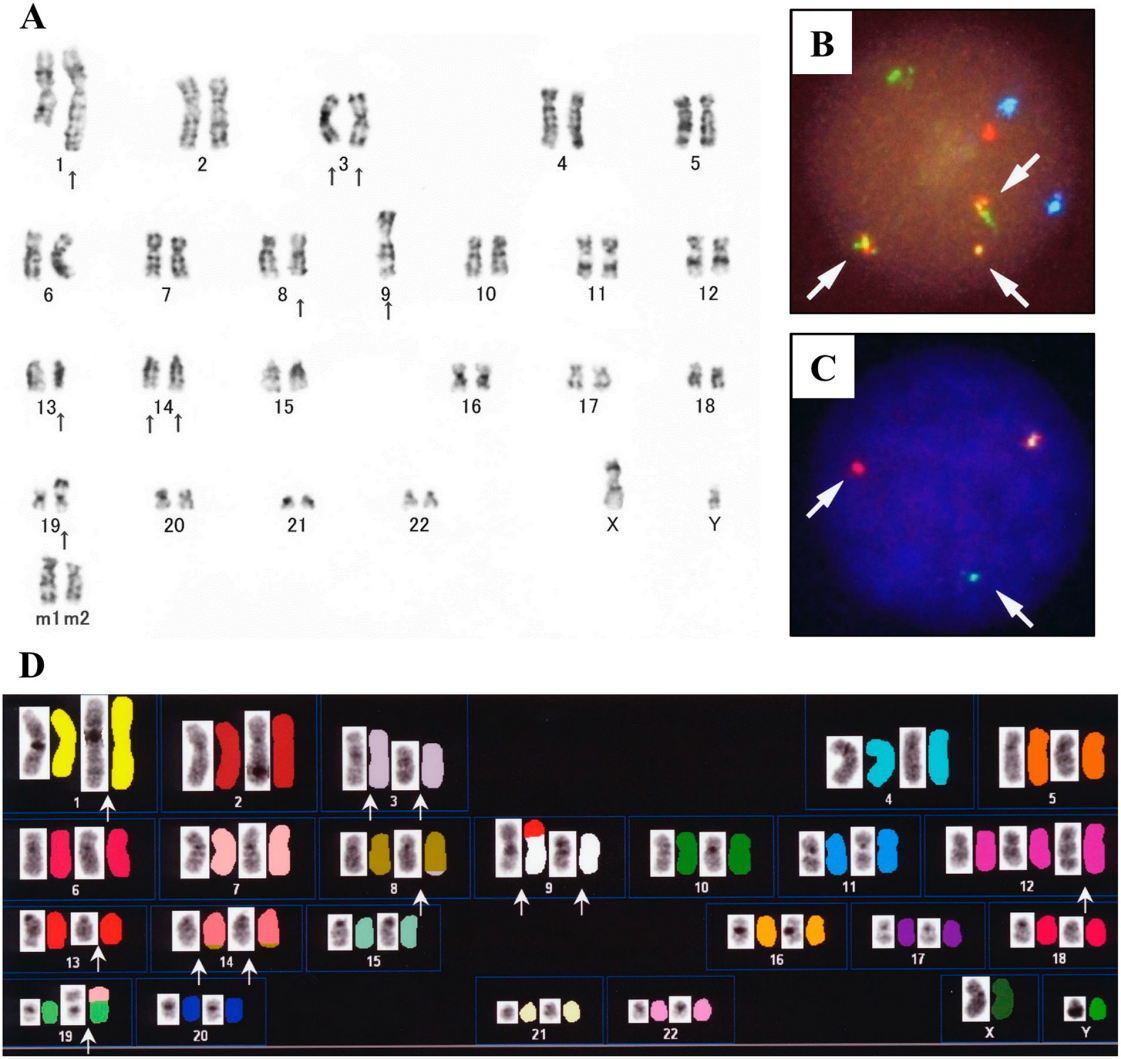
Cytogenetic analyses of DH-My6 cells **(A)** Giemsa-banding karyotype, showing the following karyotype: 47, XY, ins(1;?)(q21:?), add(3)(p25), der(3)del(3)(p?)t(3;14)(q27;q32), add(8)(q24), -9, add(9)(p11), del(13)(q?), der(14)t(8;14)(q4;q32), add(19)(p13), +mar1, +mar2. **(B)** FISH analysis with the *IGH*/*MYC* dual-color probes, showing fusion signals for the *IGH*-*MYC* gene rearrangement (arrows). The green signal corresponds to the normal *IGH* allele, red to the normal *MYC* allele, yellow to the *IGH*-*MYC* fusion gene, and blue to the centromeric region of chromosome 8. **(C)** FISH analysis for *BCL6* gene rearrangement with the break-apart probes, showing split signals for *BCL6* (arrows). Yellow signal corresponds to the intact, nonrearranged *BCL6* locus, while separate red (5' *BCL6* FISH DNA probe) and green (3' *BCL6* FISH DNA probe) signals indicate the *BCL6* rearrangement. **(D)** SKY karyotype (left side, reverse 4′,6-diamidino-2-phenylindole staining; right side, SKY), showing the following karyotype: 47, XY, der(1)(pter→q21::1?::q21→qter), del(3)(p25p26), del(3)(q27), der(8)t(3;8)(q27;q24), der(9)t(9;13)(p13;q14), inv(9)(p21q11), der(12)(pter→q24.1::12?), der(14)t(8;14)(q24;q32), der(19)t(7;19)(?;p13).

G-banding chromosomal analysis of DH-My6 cells 2 months after cell line establishment showed a complex karyotype, including der(3)t(3;14)(q27;q32) and der(14)t(8;14)(q24;q32) (Figure 2A). The karyotype showed a close resemblance to that of the primary lymphoma cells, indicating that that the DH-My6 cells were indeed derived from the primary lymphoma cells. Fluorescence *in situ* hybridization (FISH) analyses revealed the occurrence of the *IGH*–*MYC* fusion gene and the *BCL6* gene rearrangement in all DH-My6 cells analyzed (Figure 2B–2C). Spectral karyotyping (SKY) analysis of the metaphase cells showed the presence of der(14)t(8;14)(q24;q32) (Figure 2D). In addition, it revealed a cryptic translocation on der(8)t(3;8)(q27;q24). Combined G-banding and SKY analysis yielded the following karyotype: 47, XY, der(1)(pter→q21::1?::q21→qter), der(3)del(3)(p25p26)t(3;14)(q27;q32), der(8)t(3;8)(q27;q24), der(9)t(9;13)(p13;q14), inv(9)(p21q11), der(12)(pter→q24.1::12?), der(14)t(8;14)(q24;q32), der(19)t(7;19)(?;p13).

### Presence of both *BCL6*–*IGH* and *MYC*–*IGH* linkages

**Figure 3:**
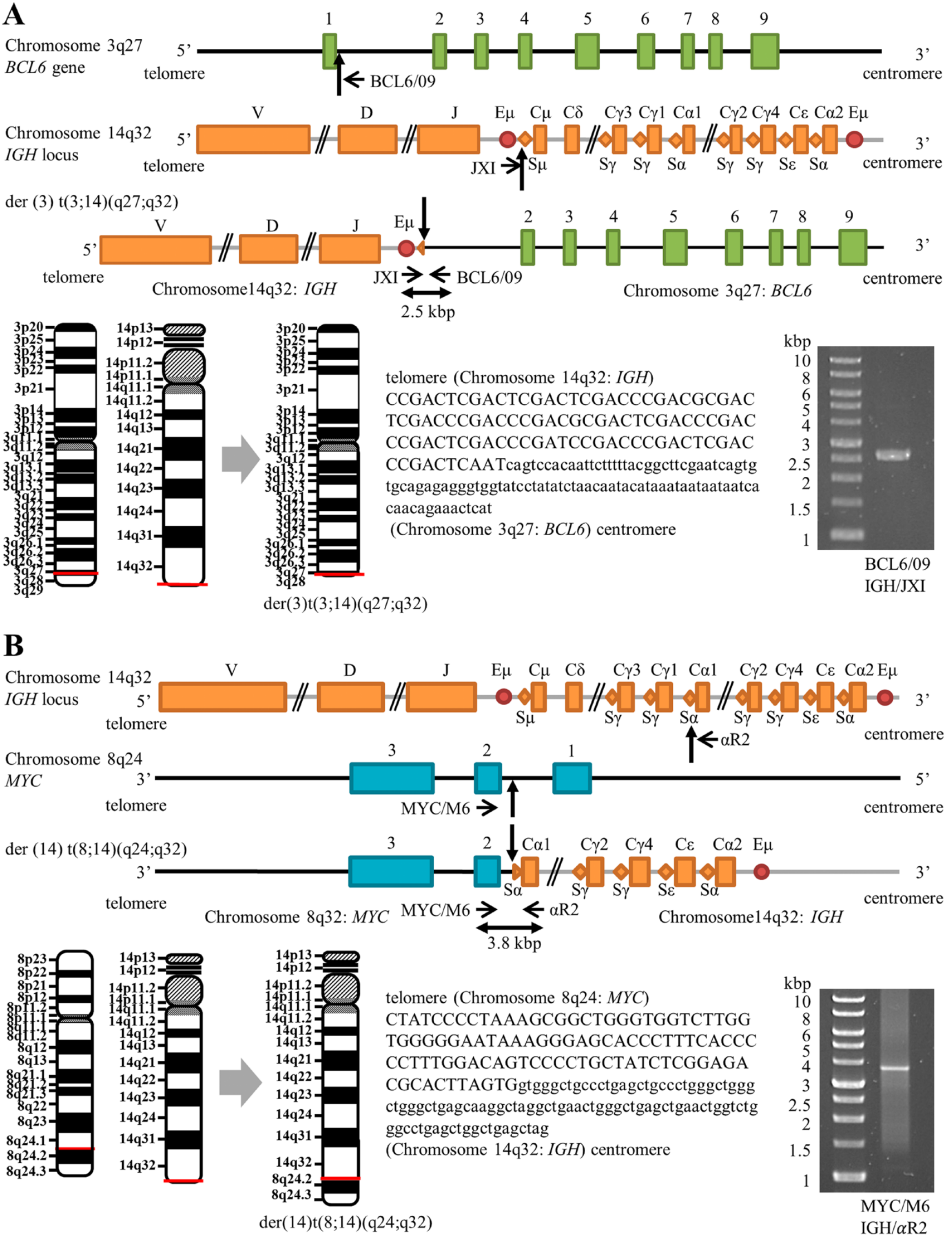
Schematic positions of *BCL6*–*IGH* and *MYC–IGH* breakpoints (not to scale) **(A)** Maps of the *BCL6* on chromosome 3, the *IGH* locus on chromosome 14, and the *BCL6–IGH* fusion gene on der(3) t(3;14)(q27;q32), showing the positions of breakpoints (vertical arrows) and positions of forward (JX1) and reverse (BCL6/09) primers (horizontal arrows). Sequence alignments of the der(3) t(3;14)(q27;q32) junction are shown with capital letters representing the *IGH* 14q32 sequence and small letters representing the *BCL6* 3q27 sequence. **(B)** Maps of the *MYC* on chromosome 8, the *IGH* locus on chromosome 14, and the *MYC–IGH* fusion gene on der(14)t(8;14)(q24;q32), showing the positions of breakpoints (vertical arrows) and positions of forward (MYC/M6) and reverse (αR2) primers (horizontal arrows). Sequence alignments of the der(14)t(8;14)(q24;q32) junction are shown with capital letters representing the *MYC* 8q24 sequence and small letters representing the *IGH* 14q32 sequence. Horizontal red lines shown on the schematic chromosomes indicate the breakage or fusion points for the chromosomal translocations. Images of ethidium bromide-stained gel electrophoresis separation of LD–PCR products are also shown. The sizes of the products are 2.5 kb and 3.8 kb for the *BCL6*–*IGH* and *MYC*–*IGH* fusion genes, respectively. Molecular weight markers are shown on the left.

Although the SKY assay did not visualize the der(3)t(3;14)(q27;q32) karyotype, we determined successfully the exact fusion positions of *BCL6*–*IGH* linkage by long–distance PCR (LD–PCR). We selected primers targeting the sequences of *BCL6* intron 1 and the *IGH* switch region, because *BCL6/IGH* translocations frequently occur at these regions in B-cell lymphomas [15, 16]. The primer BCL6/09 combined with primer JXI yielded a PCR product sized approximately 2.5 kilobase (kb), and subsequent sequence analysis confirmed fusion between the first intron of the *BCL6* gene (205 base pairs apart from the 3' end of exon 1) and the *IGH* switch region Sμ (Figure 3A). Likewise, *MYC*–*IGH* linkage as a result of der(14)t(8;14)(q24;q32) karyotype was demonstrated by LD–PCR using primers recognizing the *MYC* exon 2 (MYC/M6) and the *IGH* switch region Sɑ (ɑR2), which produced a PCR product sized approximately 3.8 kb. We found breakpoints within *MYC* intron 1 (1412 base pairs apart from the 3' end of exon 1) and the *IGH* region Sα (Figure 3B). Sequences around fusion regions on the *MYC*–*IGH* fusion gene and the *BCL6*–*IGH* fusion gene are also shown in Figure 3.

### Short tandem repeat (STR) DNA fingerprinting analysis of primary cells and DH-My6 cells

The relatedness of the primary cells and DH-My6 cells was determined by comparing their STR loci profiles. STR DNA fingerprinting analysis based on genotyping of 10 loci showed that the primary lymphoma cells and DH-My6 cells shared 100% identity (Table 1). These results demonstrated that the DH-My6 cells were indeed derived from the patient’s tumor cells.

**Table 1:** STR DNA fingerprinting of primary cells and DH-My6 cells

Sample	STR Loci									
	*TH01*	*D21S11*	*D5S818*	*D13S317*	*D7S820*	*D16S539*	*CSF1PO*	*AMEL*	*vWA*	*TPOX*
Primary cells	6, 7	30	10, 12	8	10, 12	12, 13	12	X, Y	14, 17	8
DH-My6 cells	6, 7	30	10, 12	8	10, 12	12, 13	12	X, Y	14, 17	8

**Abbreviation:** STR, short tandem repeat.

### Xenotransplantation of DH-My6 cells into nude mice

**Figure 4:**
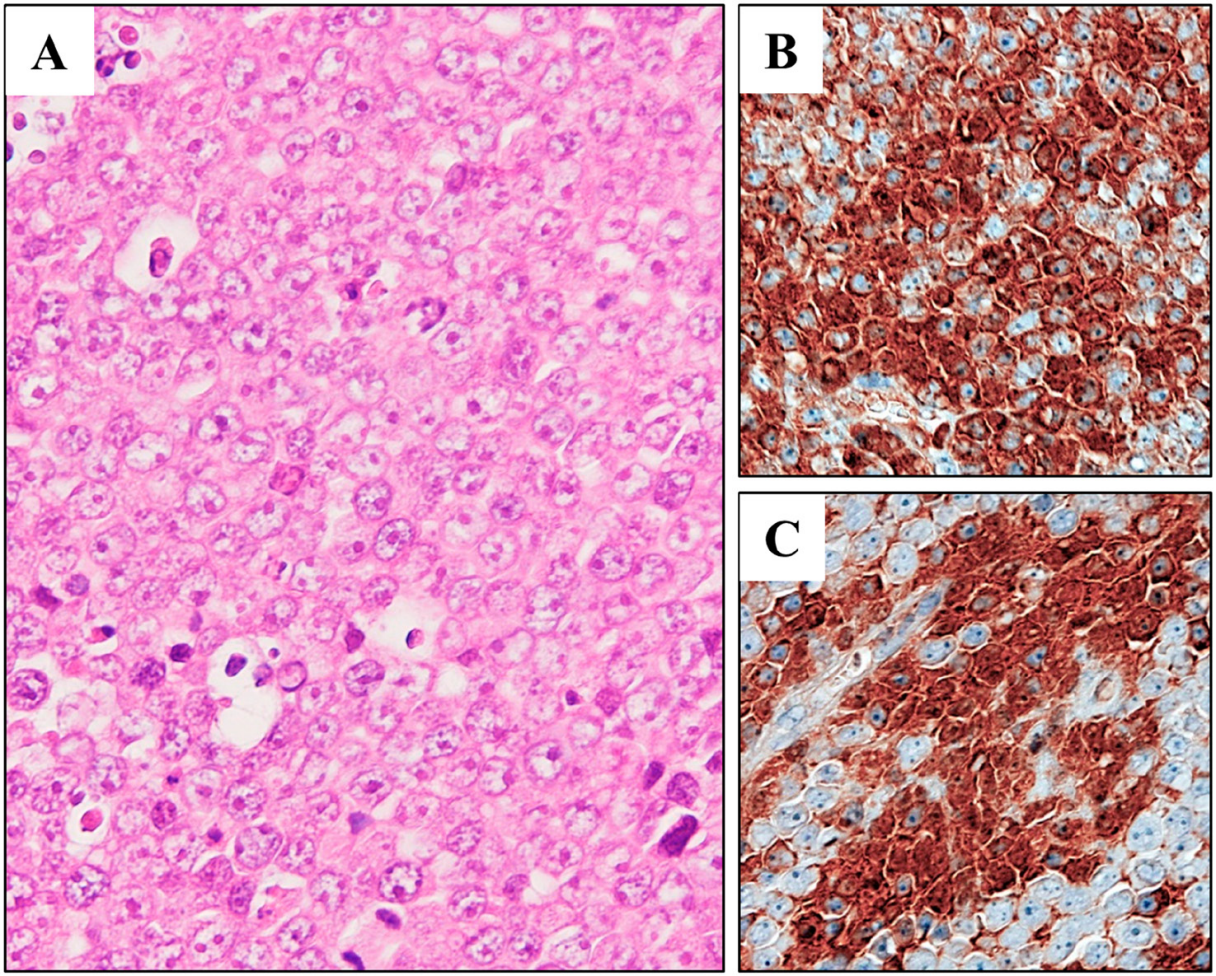
Xenotransplantation of DH-My6 cells into nude mice **(A)** Hematoxylin and eosin staining of a tumor, showing lymphoma cells with an indistinct cell border and prominent nucleoli. **(B)** Immunohistochemistry showing positive staining for MYC in a significant proportion of lymphoma cells. **(C)** Immunohistochemistry showing positive staining for BCL6 in a significant proportion of lymphoma cells.

DH-My6 cells gave rise to tumors in all nude mice tested after simultaneous inoculation. The palpable subcutaneous nodules could be detected within 3 weeks and grew larger with no sign of regression. Histology of the tumors showed diffuse infiltrations of medium-to-large-sized lymphoma cells with round or slightly irregular nuclei and one or more prominent nucleoli (Figure 4A). Immunohistochemistry showed the expressions of MYC and BCL6 (Figure 4B–4C). LD–PCR confirmed that the tumor cells carried both the *IGH*–*MYC* and *IGH*–*BCL6* fusion genes. These results indicated that the same features were maintained in this *in vivo* model.

### Antiproliferative effects of *MYC* and *BCL6* inhibitions evaluated using small interfering RNAs (siRNAs)

**Figure 5:**
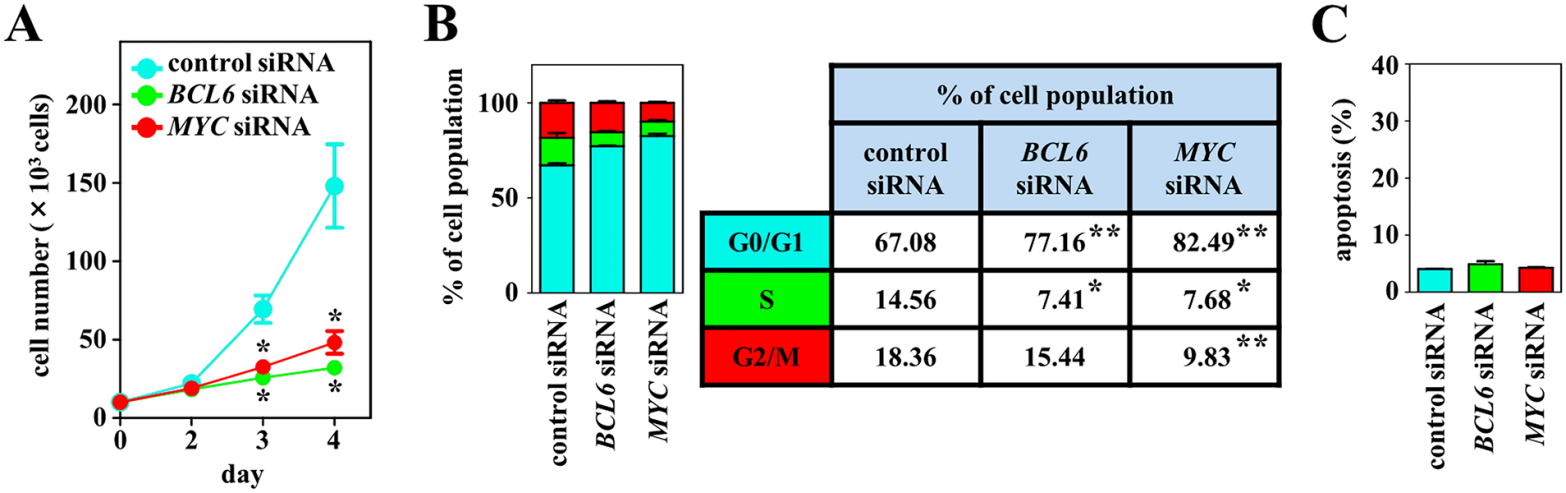
Effects of *MYC* and *BCL6* knockdown using siRNAs on cell growth, cell cycle, and apoptosis in DH-My6 cells **(A)** Cell growth assay. After transfection with *MYC*, *BCL6* or control siRNAs, viable cells were counted every 24 h. **(B)** Cell cycle analysis conducted at 48 h after transfection. Percentages of the cell population in each stage of the cell cycle are presented outside the graph. **(C)** Apoptosis assay conducted at 48 h after transfection. The graph shows the percentage of apoptotic cells in the total cell population. All experiments were repeated independently three times, and data are expressed as the mean ± SEM. Significant expression differences are shown as ^*^P < 0.05; ^**^P <0.01.

The *MYC* and *BCL6* gene expression levels were assayed by real-time quantitative reverse-transcription PCR (RT–qPCR) on the DH-My6 cell line and a panel of seven DLBCL cell lines classified as the GCB subtype. Both *MYC* and *BCL6* genes were expressed at relatively higher levels in the DH-My6 cell line (Supplementary Figure 1). Given these results, we assessed the effects of *MYC* and *BCL6* inhibitions on cell proliferation by applying gene-specific siRNAs. Transduction of each *MYC* and *BCL6* siRNA resulted in a significant decrease in cell proliferation compared with the control siRNA and caused significant increases in the proportion of cell population at the G0/G1 phase of the cell cycle, whereas they did not cause significant apoptosis (Figure 5). These results implied that the antiproliferative effects of blocking *MYC* and *BCL6* were mediated mainly through cell cycle inhibition.

### Antiproliferative effects of MYC and BCL6 inhibitors

**Figure 6:**
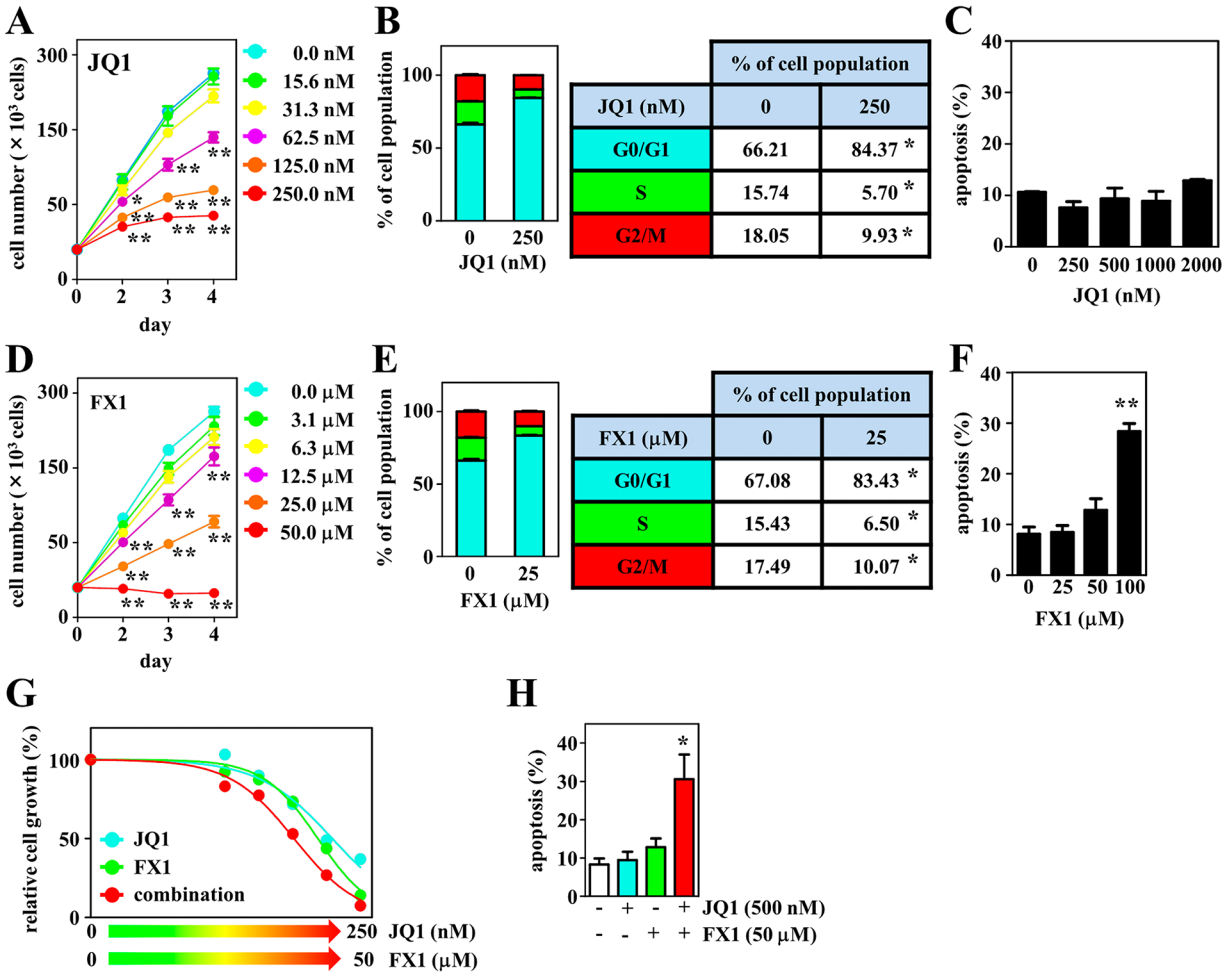
Effects of the BET inhibitor JQ1, the BCL6 inhibitor FX1, and their combination on cell growth, cell cycle, and apoptosis in DH-My6 cells **(A)** Cell growth assay following treatment with the indicated doses of JQ1. **(B)** Cell cycle analysis conducted at 48 h after treatment with JQ1 (250 nM). **(C)** Apoptosis assay conducted at 48 h after treatment with the indicated doses of JQ1. **(D)** Cell growth assay following treatment with the indicated doses of FX1. **(E)** Cell cycle analysis conducted at 48 h after treatment with FX1 (25 μM). **(F)** Apoptosis assay conducted at 48 h after treatment with the indicated doses of FX1. **(G)** Cell growth assay following treatment with the various doses of JQ1, FX1, and their combinations for 72 h. The numbers of viable cells are normalized as a percentage of the viable cell numbers of DMSO-treated controls. **(H)** Apoptosis assay conducted at 48 h after a single treatment with JQ1 (500 nM), FX1 (50 μM), and their combination at each concentration that gave a limited effect on apoptosis when treated with a single agent. Data are shown as the mean ± SEM of three independent experiments. Significant expression differences are shown as ^*^P < 0.05; ^**^P <0.01.

We next evaluated the effects of chemical inhibitors targeting MYC and BCL6 on proliferation by exposing the DH-My6 cells to them at various concentrations. In parallel with experiments on DH-My6 cells, four B-cell lymphoma cell lines with a GCB type, including two DLBCL cell lines (Su-DHL-5 and HT) and two *MYC*/*BCL2* DHL cell lines (Su-DHL-10 and Nu-DHL-1), were also tested. First, we employed 10058-F4, a direct MYC inhibitor that targets the interaction between MYC and MAX [17]. 10058-F4 significantly suppressed DH-My6 cell growth by induction of cell cycle arrest at the G0/G1 phase (Supplementary Figure 2A). Alternatively, because 10058-F4 has limited clinical utility because of its rapid degradation [17], we also treated DH-My6 cells with JQ1. This is an inhibitor targeting the bromodomain and extraterminal domain (BET) family of bromodomain (BRD) proteins, with the highest affinity for BRD4, and suppresses oncogenic transcription factors mainly through inhibiting the function of MYC [18, 19]. Like 10058-F4, JQ1 treatment significantly attenuated cell growth in a dose-responsive manner (Figure 6A). The estimated half maximal inhibitory concentration (IC_50_) at 72 h treatment in DH-My6 cells was 141 nM, which indicates a sensitivity to BET inhibition [19, 20], but was higher than the IC_50_ values in cell lines tested (Table 2). Cell cycle analysis showed an increase in the G0/G1 phase and reduction in S and G2/M phases following JQ1 (250 nM) treatment (Figure 6B). However, JQ1 treatment with concentrations as high as 2,000 nM induced cell apoptosis only weakly or not at all (Figure 6C).

**Table 2:** Estimated IC_50_ values in the lymphoma cell lines treated with inhibitors for 72 h

cell line	origin	IC_50_			
		JQ1 (nM)	FX1 (μM)	Volasertib (nM)	Vorinostat (nM)
HT	GCB-type DLBCL	55.7	21.6	7.4	630.9
Su-DHL-5	GCB-type DLBCL	48.5	8.3	5.4	468.8
Su-DHL-10	*MYC*/*BCL2* DHL	34.6	15.9	8.5	322.8
Nu-DHL-1	*MYC*/*BCL2* DHL	65.7	21.6	>10.0	608.1
DH-My6	*MYC*/*BCL6* DHL	140.6	20.9	4.6	346.7

**Abbreviation:** IC_50_, half maximal inhibitory concentration.

We also explored the antitumor effects of two BCL6 inhibitors, 79-6 and FX1, both of which bind to the groove of the BCL6 BTB domain and disrupt *BCL6* transcriptional complexes [21, 22]. These agents led to significant suppression of DH-My6 cell growth in a dose-dependent manner (Supplementary Figure 2B and Figure 6D). FX1 showed a stronger antiproliferative effect (FX1, IC_50_ 21 μM vs. 79-6, IC_50_ >100 μM), consistent with previous findings that FX1 exhibits 10-fold greater inhibitory activity against BCL6 BTB domain than 79-6 [22, 23]. FX1 treatment (25 μM) induced cell cycle arrest at the G0/G1 phase (Figure 6E), but significant apoptosis was not elicited with concentrations as high as 50 μM (Figure 6F). Taken together, consistent with genetic inhibition, single chemical inhibition targeting MYC and BCL6 induced cell cycle arrest consistently but led to only limited apoptosis.

Next, we assessed the effects of combination treatments with the BET and BCL6 inhibitors. DH-My6 cells were treated with JQ1 plus FX1, and the efficacy was compared with that of the single agent. Cotreatment showed an additive inhibitory effect on DH-My6 cell growth with a combination index (CI) value of 1.0 (Figure 6G). Notably, apoptosis was strikingly induced in combination with JQ1 (500 nM) and FX1 (50 μM) at each concentration that gave a limited effect on apoptosis when treated with single agents (Figure 6H). These data imply that dual inhibition of MYC and BCL6 produced better antigrowth efficacy than the single agent.

The estimated IC_50_ values of these agents for other cell lines tested in this study are shown in Table 2.

### Agents leading to the effective inhibition of tumor cell growth

**Figure 7:**
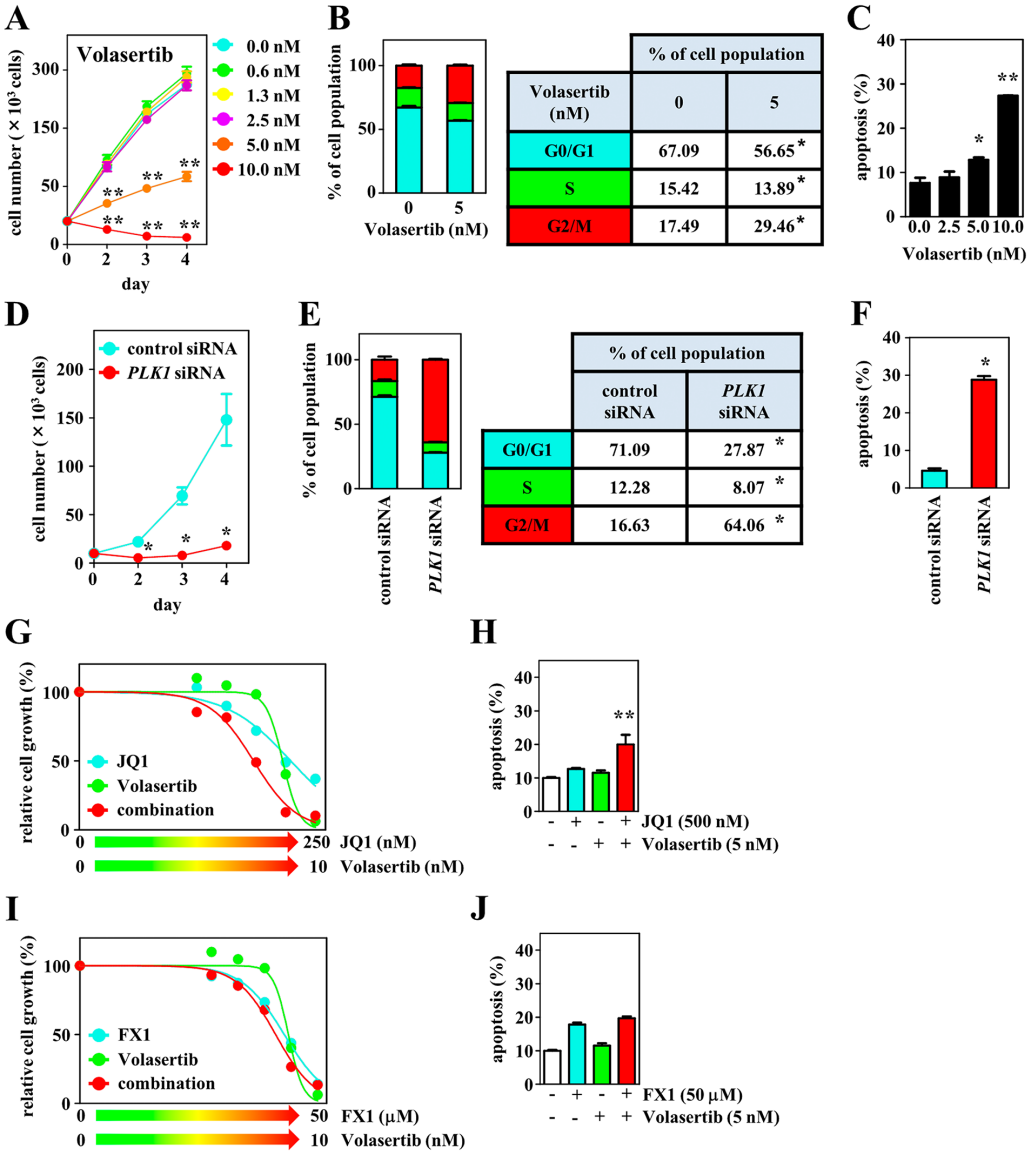
Effects of PLK1 interruption and its combination with BET or BCL6 inhibition on cell growth, cell cycle, and apoptosis in DH-My6 cells **(A)** Cell growth assay following treatment with the indicated doses of volasertib. **(B)** Cell cycle analysis conducted at 48 h after treatment with volasertib (5 nM). **(C)** Apoptosis assay conducted at 48 h after treatment with the indicated doses of volasertib. **(D)** Cell growth assay following transfection with *PLK1* or control siRNAs. **(E)** Cell cycle analysis conducted at 48 h after siRNA transfection. **(F)** Apoptosis assay conducted at 48 h after siRNA transfection. **(G)** Cell growth assay following treatment with the various doses of volasertib, JQ1, and their combinations for 72 h. **(H)** Apoptosis assay conducted at 48 h after a single treatment with volasertib (5 nM), JQ1 (500 nM), and their combination at doses that did not induce significant apoptosis individually. **(I)** Cell growth assay following treatment with the various doses of volasertib, FX1, and their combinations for 72 h. **(J)** Apoptosis assay conducted at 48 h after a single treatment with volasertib (5 nM), FX1 (50 μM), and their combination at doses that did not induce significant apoptosis individually. Data are shown as the mean ± SEM of three independent experiments. Significant expression differences are shown as ^*^P < 0.05; ^**^P <0.01.

We explored the inhibitory effects of a chemotherapeutic agent that targets cell cycle regulation in combination with JQ1 or FX1. Because PLK1 is a master regulator of several cell cycle events [24, 25], we investigated its antitumor effects, using the selective PLK1 inhibitor volasertib [26]. *PLK1* expression was detected in DH-My6 cells with expression levels similar to the DLBCL cell lines tested, except for Nu-DHL-1 in which lower levels of *PLK1* were detected (Supplementary Figure 1). Treatment with volasertib at concentrations of 5.0 nM or higher resulted in a drastic reduction of DH-My6 cell proliferation (Figure 7A). The estimated IC_50_ was 4.6 nM, lower than in the *MYC*/*BCL2* DHL cell lines tested in this study (Su-DHL-10, 8.5 nM; Nu-DHL-1, >10.0 nM; Table 2) and than in a panel of various cancer cell lines reported previously (11–37 nM) [27]. Exposure of DH-My6 cells to volasertib caused a significant increase in the proportion of cells at the G2/M phase and also induced a marked increase in apoptosis (Figure 7B–7C). Genetic inhibition of *PLK1* through siRNA knockdown also diminished cell proliferation significantly over time relative to control siRNA and produced G2/M cell cycle arrest and significant apoptosis in DH-My6 cells (Figure 7D–7F). Thus, PLK1 interruption by the chemical inhibitor and by siRNA knockdown approaches both showed efficient antitumor activity.

When volasertib was combined with JQ1 or FX1, a synergistic antiproliferative effect was given only with the combination of volasertib with JQ1 with a CI value of 0.9 (Figure 7G–7J). This combination also led to a greater level of apoptosis than the use of single agents. These results suggest that coinhibition of PLK1 and BET exerted greater antitumor effects through blocking the cell cycle at both G0/G1 and G2/M phases and through the induction of apoptosis.

**Figure 8:**
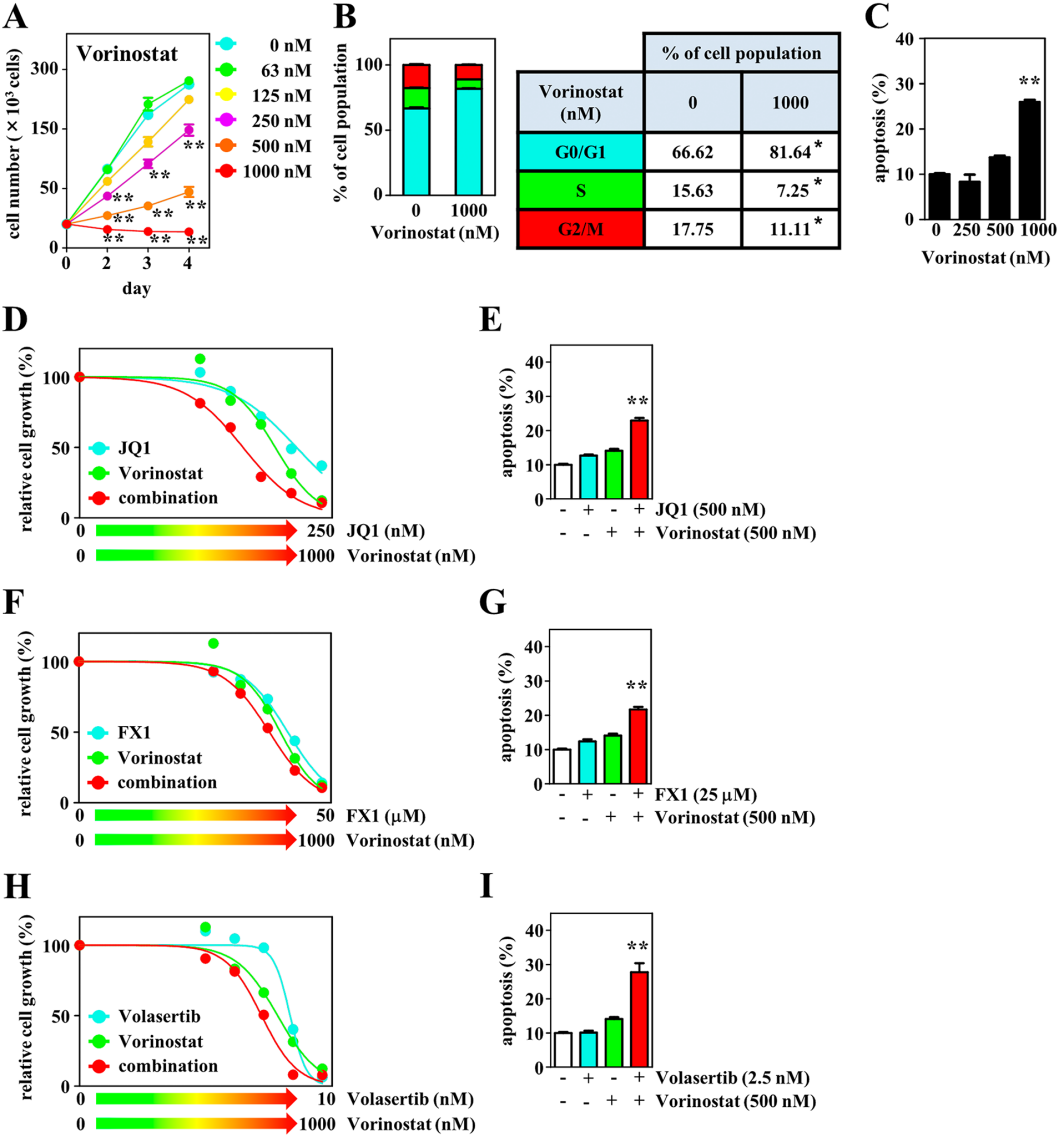
Effects of the HDAC inhibitor vorinostat and its combination with JQ1, FX1, or volasertib on cell growth, cell cycle, and apoptosis in DH-My6 cells **(A)** Cell growth assay following treatment with the indicated doses of vorinostat. **(B)** Cell cycle analysis conducted at 48 h after treatment with vorinostat (1 μM). **(C)** Apoptosis assay conducted at 48 h after treatment with the indicated doses of vorinostat. **(D)** Cell growth assay following treatment with the various doses of vorinostat, JQ1, and their combinations for 72 h. **(E)** Apoptosis assay conducted at 48 h after a single treatment with vorinostat (500 nM), JQ1 (500 nM), and their combination at doses that did not induce significant apoptosis individually. **(F)** Cell growth assay following treatment with the various doses of vorinostat, FX1, and their combinations for 72 h. **(G)** Apoptosis assay conducted at 48 h after single treatment with vorinostat (500 nM), FX1 (25 μM), and their combination at doses that did not induce significant apoptosis individually. **(H)** Cell growth assay following treatment with the various doses of vorinostat, volasertib, and their combinations for 72 h. **(I)** Apoptosis assay conducted at 48 h after a single treatment with vorinostat (500 nM), volasertib (2.5 nM), and their combination at doses that did not induce significant apoptosis individually. Data are shown as the mean ± SEM of three independent experiments. Significant expression differences are shown as ^*^P < 0.05; ^**^P <0.01.

In addition, we evaluated the antitumor effect of HDAC inhibition using the selective HDAC inhibitor vorinostat as a single agent and in combination with BET or BCL6 inhibitors. Vorinostat significantly suppressed DH-My6 cell growth in a dose-responsive manner (Figure 8A). Vorinostat blocked cell cycle progression at the G0/G1 phase (Figure 8B) and induced pronounced apoptosis (Figure 8C). The estimated IC_50_ was 350 nM. Treatment of DH-My6 cells with vorinostat in combination with JQ1 resulted in synergistic inhibition of cell growth with a CI value of 0.7 (Figure 8D) and led to more efficient apoptosis than treatment with the single agents (Figure 8E). The combination of vorinostat (500 nM) with FX1 (25 μM) also resulted in greater apoptosis (Figure 8F–8G). This effect could be achieved at doses which did not induce significant apoptosis with individual agents.

HDAC and PLK1 inhibitors exhibit complementary mechanisms of actions [28, 29]. Therefore, we also studied the antitumor effect of a combination of vorinostat with volasertib. This showed a better growth inhibitory effect than the single agents at lower concentrations (Figure 8H). Exposure of DH-My6 cells to a low concentration of volasertib (2.5 nM) in conjunction with vorinostat (500 nM) induced a dramatic increase in apoptosis, although each agent individually had no significant apoptotic effect (Figure 8I).

## DISCUSSION

Features of the DH-My6 cell line are summarized in Table 3. This well-characterized DH-My6 cell line has several strengths. *MYC*/*BCL6* DHL cases make up only 8% of all DHL/THL cases [1, 4]. Moreover, there is concern that many of the reported *MYC*/*BCL6* DHL cases may actually represent THL [1]. Clinicopathologically, THL cases resemble *MYC*/*BCL2* DHL cases more closely than *MYC*/*BCL6* DHL cases [30]. Consequently, establishment of DHL cell lines that only have *MYC* and *BCL6* translocations is meaningful in that they offer the possibility of analyzing the effects of such unique rearrangements in more detail. The DH-My6 cell line presented here is a new one derived from a “pure *MYC*/*BCL6* DHL” case with *MYC* and *BCL6* translocations. Its derivation from the patient was clearly authenticated by our STR analysis. DH-My6 cells with morphologic features of DLBCL are of the GCB type, which is an immunophenotype observed in 50–80% of published *MYC*/*BCL6* DHL cases [9, 10, 31, 32]. DH-My6 cells showed only faint CD20 expression, as with the pretreated primary lymphoma cells, eliminating the effect of rituximab as a cause of this unique finding. Based on reviews in the literature [14, 33, 34], most of the *MYC*/*BCL2* DHL cases showed decreased expression of CD20. Although mechanisms resulting in decreased expression of CD20 after rituximab treatment have been studied using rituximab-resistant lymphoma cell lines and the results have suggested the association of epigenetic or genetic changes with downregulation of CD20 [35, 36], the underlying mechanism for diminishing CD20 expression in *de novo* DHL cells has not been investigated. Clinically, the decreased expression of CD20 results in the loss of efficiency of anti-CD20 antibody therapies. The DH-My6 cell line will also be a useful tool for the elucidation of the mechanism of CD20 downregulation in DHL. Moreover, the DH-My6 cells show strong CD38 expression, which appears to be a characteristic phenotype of lymphomas with *MYC* rearrangement [34, 37]. Because a satisfactory screening for rare cases of *MYC*/*BCL6* DHL has not been defined so far [5], these flow cytometric findings might be helpful in triaging lymphomas for confirmatory targeted FISH analysis.

**Table 3:** Synopsis of data on the DH-My6 cell line

Parameter	Features
Clinical data	
Patient	54-year-old man
Diagnosis	HGBL with *MYC* and *BCL6* rearrangements
Treatment status	At diagnosis
Specimen Year of establishment	Bone marrow 2010
Cell line	
Culture medium	RPMI1640 + 10% FCS
Growth pattern	Single cells in suspension
Doubling time	20 h
Optimal cell density	5 × 10^5^ cells/ml
Optimal split	1:3 every 3–4 days
Cryopreservation	In 80% medium, 10% FCS, 10% DMSO
Morphology	medium-to-large-sized cells with round or slightly irregular nuclei
Viral status	Negative for Epstein–Barr virus
Immunoprofiles	Strongly positive for CD10, CD19, CD22, CD38; Weekly positive/negative for CD20
Karyotypic analysis in conjunction with SKY	47, XY, der(1)(pter→q21::1?::q21→qter), der(3)del(3)(p25p26)t(3;14)(q27;q32), der(8)t(3;8)(q27;q24), der(9)t(9;13)(p13;q14), inv(9)(p21q11), der(12)(pter→q24.1::12?), der(14)t(8;14)(q24;q32), der(19)t(7;19)(?;p13)
Tumorigenic capacity	Subcutaneous growth in BALB/c nude mice
Authentication	Yes (by STR DNA fingerprinting and cytogenetic characteristics)

**Abbreviations:** HGBL, high-grade B-cell lymphoma; STR, short tandem repeat.

Another important characteristic of DH-My6 is that the cells carry gene rearrangements with concurrent t(8;14)(q24;q32)/*MYC*–*IGH* and t(3;14)(q27;q32)/*BCL6*–*IGH* linkages. Our SKY analysis detected der(14)t(8;14)(q24;q32) and cryptic der(8)t(3;8)(q27;q24) but not der(3)t(3;14)(q27;q32). It is probable that the chromosome 3 with deletion q27 detected by SKY was actually a der(3)t(3;14)(q27;q32), because SKY usually cannot visualize the small segment 14q32→14qter on a der(3)t(3;14)(q27;q32) background [38]. In fact, LD–PCR assays readily detected both the 3' *BCL6*–5' *IGH* fusion sequence in the *BCL6* intron 1 on chromosome 3 and the 3' *MYC*–3' *IGH* fusion sequence in the *MYC* intron 1 on chromosome 14.

DH-My6 cells had higher expression levels of both *MYC* and *BCL6*. Ueda *et al.* [39] showed that *BCL6* is significantly upregulated when its translocation partner is an *IG* gene in DLBCL. Wilda *et al.* [40] demonstrated that the level of *MYC* overexpression is dependent on breakpoint location within the *MYC* locus and that significant higher *MYC* expression is associated with breakpoint from exon 1 to intron 1. These events, as observed in DH-My6 cells, are likely to lead to higher levels of both *MYC* and *BCL6* in this cell line. Several studies have shown that most patients with *MYC*/*BCL6* DHL have an aggressive clinical course [9, 10, 32]. In contrast, in other studies, patients with *MYC* and *BCL6* rearrangements were not necessarily associated with an inferior prognosis [11, 41]. A possible explanation for the discrepancy is patient selection. These previous reports on the prognostic value of *MYC*/*BCL6* DHL included *MYC*/*BCL6* DHL cases of a non-GCB type, which have a tendency to a better prognosis than the GCB type that is observed almost exclusively in *MYC*/*BCL2* DHL cases [41]. In addition, such studies did not assess whether the specific *BCL6* partner is an *IG* or a non-*IG* gene and might include t(3;8)(q27;q24) with simultaneous rearrangement of *BCL6* and *MYC* in the context of a *MYC*/*BC6* double hit. Indeed, the most common non-*IG/BCL6* partner is *MYC* [9], and the t(3;8)(q27;q24) karyotype is frequently observed in cases of “multiple-hit” lymphoma [42–44], as shown in DH-My6 cells. It should be noted, however, that t(3;8)(q27;q24) represents a *MYC*/non-*IG* single hit activating solely *MYC* [44, 45]. Thus, *MYC*/*BCL6* DHL appears to be heterogeneous group of tumors consisting of cases with both favorable and poor prognoses. The latter cases are resistant to standard frontline therapy for DLBCL and might require specific therapeutic approaches. We believe that the DH-My6 cell line carrying concurrent t(8;14)(q24;q32) and t(3;14)(q27;q32) and the higher expression of both MYC and BCL6 will also prove to be a valuable experimental tool to find potential therapeutic targets against aggressive *MYC*/*BCL6* DHLs.

In this study, we demonstrated that targeted inhibitions of MYC by 10058-F4 and BCL6 by its inhibitors (79-6 and FX1) resulted in a significant attenuation of DH-My6 cell growth through induction of G0/G1 cell cycle arrest. Similar results were obtained when the antiproliferative effect was monitored by genetic inhibition using *MYC* and *BCL6* gene-specific siRNAs. Although the importance of MYC in carcinogenesis is well documented, therapies targeting MYC directly are not available currently. Accordingly, the BET inhibitor JQ1 has been used as a therapeutic strategy to target MYC [18]. In one study, a panel of seven GCB-type DLBCL cell lines, including two *MYC*/*BCL2* DHL cell lines (Su-DHL-10 and OCI-Ly18), were treated with JQ1, and all showed decreased cell growth [20]. Another study has demonstrated that the BCL2 inhibitor venetoclax enhanced the antiproliferative activity when it was combined with JQ1 in two *MYC*/*BCL2* DHL cell lines (Sc-1 and OCI-Ly18) [46]. Because BCL6 is required for the survival of certain types of lymphoma, it is also conceivable that BCL6 inhibitors might prove to be useful for *MYC*/*BCL6* DHL [21]. In the present study, a combination of JQ1 and FX1 showed a better inhibitory effect on DH-My6 cell growth. Notably, pronounced apoptosis was induced when these compounds were combined. These results suggest that cotargeting of MYC and BCL6 is likely beneficial for antitumor activity in *MYC*/*BCL6* DHL cells with high expression levels of *MYC* and *BCL6*. Indeed, aggressive *MYC*/*BCL6* DHL tumors showed a trend toward higher *MYC* mRNA expression compared with *MYC*/*BCL2* DHL [31]. Thus, as a proof-of-principle, our findings provide a rationale for preclinical trials on the efficacy of BRD4 inhibitors such as JQ1 in combination with a BCL6 inhibitor for the development of valid treatment modalities for patients with aggressive *MYC*/*BCL6* DHL.

In an effort to identify other therapeutic targets, we also demonstrated that the PLK1 inhibitor volasertib alone significantly impaired the viability of DH-My6 cells. Notably, we found synergistic antitumor effects when volasertib was combined with JQ1 by blocking the cell cycle at both G2/M and G0/G1 phases. Murga-Zamalloa *et al.* [47] showed that MYC and PLK1 expression levels were strongly correlated in cases of aggressive DLBCLs and that PLK1 expression was most prevalent in “double hit” HGBC with a *MYC* translocation. Because PLK1 has been implicated in molecular cross talk with MYC [48] and volasertib possibly has an inhibitory activity on the BRD4 protein [26], it is plausible that therapeutic targeting of PLK1 in combination with JQ1 might yield a more favorable therapeutic index in MYC-associated lymphomas. Moreover, cotreatment with the HDAC inhibitor vorinostat and JQ1 synergistically reduced tumor growth in DH-My6 cells. Bhadury *et al.* [49] indicated that BET and HDAC inhibitors have similar target genes and biological effects, thus synergizing to kill MYC-induced murine lymphomas. BET and HDAC coinhibition have been also proved to be effective against human acute myelogenous leukemia cells [50]. In the present study, we also found that the combination of vorinostat and volasertib in DH-My6 cells resulted in marked apoptosis at doses that exerted little antiproliferative effect or only induced cell cycle arrest when used individually. This combination treatment appears to be effective in HGBL irrespective of *BCL2* or *BCL6* rearrangements because exposure of *MYC*/*BCL2* DHL cells (OCI-Ly18 and Carnaval) to volasertib in combination with the HDAC inhibitor belinostat increased cell death [29]. Furthermore, combination of vorinostat with FX1 significantly induced apoptosis in DH-My6 cells. Cotargeting of BCL6 and HDAC seems to be another attractive approach for efficient eradication of *MYC*/*BCL6* DHL cells. This combination has yielded more potent antilymphoma effects in BCL6-dependent DLBCL cell lines [51].

In summary, we present a novel “double hit” high-grade B-cell lymphoma cell line, designated DH-My6, with concurrent *MYC*/*IGH* and *BCL6*/*IGH* rearrangements. This well-characterized *MYC*/*BCL6* DHL cell line will provide useful models for biological studies of the less common DHL tumors. We also provide here a basis for a rational combination therapeutic strategy targeting MYC and BCL6, and in combination with PLK1 or HDAC inhibitors against aggressive *MYC*/*BCL6* DHL tumors, which will promote further *in vivo* testing in a preclinical model.

## MATERIALS AND METHODS

### Case history and cell culture

The DH-My6 cell line was established from a 54-year-old man who was referred to our hospital for treatment of a bulky retroperitoneal tumor. Pathology of a bone marrow aspiration showed diffuse infiltration of medium-to-large-sized lymphoma cells with expression of both MYC and BCL6 proteins. Chromosomal analysis showed a complex karyotype, including der(3)t(3;14)(q27;q32) and der(14)t(8;14)(q24;q32). FISH analysis revealed concurrent *MYC* and *BCL6* rearrangements. These pathology findings are shown in Supplementary Figure 3. The cells were strongly positive for CD10, CD19, CD22, and CD38, and weakly positive for CD20, and exhibited high positivity for Ki-67 staining (85–90%). The cells had a GCB immunophenotype. The patient was diagnosed as having an HGBL with *MYC* and *BCL6* rearrangements. He was treated initially with one cycle of hyperfractionated cyclophosphamide, vincristine, doxorubicin, and dexamethasone (hyper CVAD) and two cycles of cyclophosphamide, vincristine, doxorubicin, methotrexate, ifosfamide, etoposide, cytarabine, and rituximab (R-CODOX-M/R-IVAC). However, the disease relapsed immediately, and he died of disease progression 10 months after the initial presentation. Both chromosomal translocations t(3;14)(q27;q32) and t(8;14)(q24;q32) were consistently detected three times during the disease’s progression.

A heparinized bone marrow sample was obtained when his bone marrow aspirate contained 92% lymphoma cells. The cells were cultured in RPMI 1640 medium supplemented with 20% heat-inactivated FCS without any external stimulation. The cultures were incubated at 37°C in a humidified atmosphere of 5% CO2 in air and fed every 3 days by partial medium change.

This study was approved by the Ethics Committee of Kochi Medical School, Kochi University. Written informed consent was obtained from the patient before the patient died, and all experiments were performed in accordance with the regulations of the institutional review board.

### Immunophenotypic, immunohistochemical, cytogenetic, and FISH analyses

Expression of cell surface antigens was studied with fluorescein isothiocyanate-conjugated monoclonal antibodies using flow cytometry. To detect MYC and BCL6 proteins, immunohistochemistry was performed in formalin-fixed paraffin wax-embedded tissue sections using mouse monoclonal antibodies 9E10 (Santa Cruz Biotechnology, Dallas, TX, USA) and D-8 (Santa Cruz Biotechnology), respectively, based on an indirect biotin-avidin system using a biotinylated universal secondary antibody and diaminobenzidine substrate with hematoxylin counterstaining. The specificity of staining with the primary antibodies was controlled by testing the isotype-matched control antibodies in parallel. Metaphase chromosome spreads were G-banded according to standard procedures. Karyotypes have been described according to the International System for Cytogenetic Nomenclature. For SKY analysis, a probe cocktail containing 24 uniquely labeled chromosome-specific painting probes was hybridized to metaphase chromosomes according to described methods [52]. FISH analysis was performed on interphase nuclei using commercially available probes, as described previously [53, 54]. The *IGH*–*MYC* rearrangement [t(8:14)] probe is a dual-color, dual-fusion probe that detects the juxtaposition of the *IGH* locus at 14q32 to the *MYC* sequence at 8q24. To detect the *BCL6* rearrangement, a dual-color, break-apart probe flanking the major breakpoint region of *BCL6* was used.

### LD–PCR

LD–PCR was carried out as described previously with modifications [55]. Genomic DNA was extracted by the *phenol–chloroform method.* The LD–PCR reaction mixture contained 200 ng of extracted DNA, reaction buffer, a dNTP mixture, 0.2 μM of each primer, and 1.25 units of PrimeSTAR GXL DNA Polymerase (TaKaRa Bio, Shiga, Japan). To determine the fusion position of the *MYC*–*IGH* [t(8;14)] sequence, we used primers targeting the *MYC* exon 2 region, named MYC/M6 and targeting the *IGH* switch region, named αR2 [55, 56]. To determine the fusion position of the *BCL6*–*IGH* [t(3:14)] sequence, we used primers targeting the *BCL6* exon 1 region, named BCL6/09 and the *IGH* switch region, named JXI [56, 57]. The primer sequences are listed in Supplementary Table 1. The LD–PCR conditions were 2 min at 98°C, followed by 35 cycles of 10 s at 98°C, 15 s at 60°C, and 5 min at 68°C, with a final extension for 5 min at 68°C. The PCR products were analyzed by 0.8% agarose gel electrophoresis and visualized under an ultraviolet transilluminator after staining with ethidium bromide. The purified PCR products were sequenced directly, as described previously [58].

### STR DNA fingerprinting

Genomic DNA was isolated from the primary lymphoma cells and DH-My6 cells, using NucleoSpin Tissue kits (TaKaRa Bio). STR DNA fingerprinting was carried out using a GenePrint 10 System (Promega, Madison, WI, USA), which allows coamplification and detection of 10 human loci (*TH01*, *D21S11*, *D5S818*, *D13S317*, *D7S820*, *D16S539*, *CSF1PO*, *Amelogenin*, *vWA*, and *TPOX*).

### *In vivo* studies in mice

Five-week-old male BALB/c Slc-*nu*/*nu* mice (Japan SLC, Hamamatsu, Japan) were engrafted subcutaneously with 2 × 10^7^ cells in the flanks. The mice were maintained in a temperature-controlled (23 ± 2°C) and humidity-controlled (55 ± 10%) room under a constant day-night photoperiod and observed regularly for appearance of tumors. After tumor-bearing mice had been euthanized, tumors were resected surgically and subjected to pathological studies. All experimental protocols were approved by our Institutional Animal Care and Use Committee in compliance with our institutional guidelines on the care and use of animals for scientific purposes.

### Real-time RT–qPCR

Real-time RT–qPCR was used to study expression levels of the *BCL6* and *MYC* genes. Total RNA was extracted using TRIzol reagent (Thermo Fisher Scientific, Waltham, MA, USA) and RNeasy Mini kits (QIAGEN, Tokyo, Japan). Total RNA aliquots were treated with DNase to avoid any amplification of genomic DNA and were reverse-transcribed, using SuperScript VILO cDNA Synthesis kits (Thermo Fisher Scientific). An aliquot of each cDNA was subjected to qPCR analysis. The reaction was conducted on a StepOnePlus thermocycler (Thermo Fisher Scientific) with THUNDRBIRD SYBR qPCR mix (TOYOBO, Osaka, Japan) containing 0.3 μM of each primer. *PLK1* expression in DH-My6 cells was also examined as described previously [35]. The primer sequences used to determine the gene expressions are listed in Supplementary Table 1. The PCR conditions were 1 min at 95°C, followed by 40 cycles of 15 s at 95°C and 1 min at 62°C. Relative gene expression levels were calculated using 2^–ΔCt^ values, with the *β-actin* gene (*ACTB*) gene used as a housekeeping control. These experiments were performed in triplicate.

### Drug exposure

Cells were treated with a single drug or a combination of two drugs for 4 days. The MYC inhibitor 10058-F4 was purchased from Cayman Chemical (Ann Arbor, MI, USA). The BCL6 inhibitors 79-6 and FX1 were from Merck KGaA (Darmstadt, Germany) and Selleck Chemical (Houston, TX, USA), respectively. The BET domain inhibitor JQ1 was obtained from Merck KGaA, the HDAC inhibitor vorinostat was from Cayman Chemical, and the PLK1 inhibitor volasertib was from ChemScene (Monmouth Junction, NJ, USA). All reagents were dissolved in DMSO. DMSO concentration in the control medium was the same as that used to make up the highest concentration of drugs in growth medium for the same set of experiments.

### Genetic inhibition through siRNA

Cells were transfected with siRNAs on Nucleofector (Lonza, Basel, Switzerland), using C solution and the D-23 program. Stealth RNAi siRNAs directed against *MYC* (HSS181389), *BCL6* (HSS100968), and *PLK1* (HSS108120) and nontargeting control siRNA (12935-145) were obtained from Thermo Fisher Scientific. These experiments were performed in triplicate.

### Cell proliferation, apoptosis, and cell cycle analyses

For cell proliferation assays, cells were seeded in 96-well plates (2 × 10^4^ cells/well), and viable cells were counted every 24 h on a FACSCalibur flow cytometer (Becton Dickinson, Mountain View, CA, USA) by gating out cells stained with propidium iodide. To compare the antiproliferation effect of single-agent treatments with combination treatments, synergy levels were determined from CI ranges, using the Chou–Talalay method [59]: no synergism, CI > 1.1; additive effect, CI = 1.0–1.1; and synergism, CI < 1.0. For apoptosis analysis, cells were stained with annexin V-phycoerythrin and 7-amino-actinomycin D as described [35]. For cell cycle analysis, cells were fixed in cold 70% ethanol, treated with RNase, and stained with propidium iodide. Cells were analyzed using a FACSCalibur, and all flow cytometry data were analyzed using CellQuest Pro software (Becton Dickinson). These experiments were performed in triplicate.

### Statistical analysis

Data are presented as the mean ± the standard error of the mean (SEM). Differences between two groups were analyzed using the Mann–Whitney nonparametric *U* test when only two groups were compared, or by one-way analysis of variance when more than two groups were compared. A statistically significant difference was defined as *P* < 0.05.

## SUPPLEMENTARY MATERIALS FIGURES AND TABLE



## Abbreviations

DHL: double-hit lymphoma
HGBL: high-grade B-cell lymphoma
THL: triple-hit lymphoma
DLBCL: diffuse large B-cell lymphoma
IGH: immunoglobulin heavy chain
PLK1: polo-like kinase 1
HDAC: histone deacetylase
FCS: fetal bovine serum
DMSO: dimethylsulfoxide
GCB: germinal center B-cell like
FISH: fluorescence *in situ* hybridization
SKY: spectral karyotyping
LD–PCR: long-distance polymerase chain reaction
STR: short tandem repeat
RT–qPCR: quantitative reverse-transcription PCR
siRNA: small interfering RNA
BET: bromodomain and extraterminal domain
BRD: bromodomain
IC_50_: half maximal inhibitory concentration
CI: combination index
SEM: standard error of the mean
