# Supplementary material for: Generation and characteristics of a novel “double-hit” high grade B-cell lymphoma cell line DH-My6 with MYC/IGH and BCL6/IGH gene arrangements and potential molecular targeted therapies
Source: Oncotarget. 2018 Sep 11;9(71):33482–99. doi: 10.18632/oncotarget.26060 (PMC6173362; doi:10.18632/oncotarget.26060)
Supplement: Supplementary file 1 [file oncotarget-09-33482-s001.pdf]

Generation and characteristics of a novel “double-hit” high grade B-cell lymphoma cell line DH-My6 with *MYC/IGH* and *BCL6/IGH* gene arrangements and potential molecular targeted therapies

SUPPLEMENTARY MATERIALS

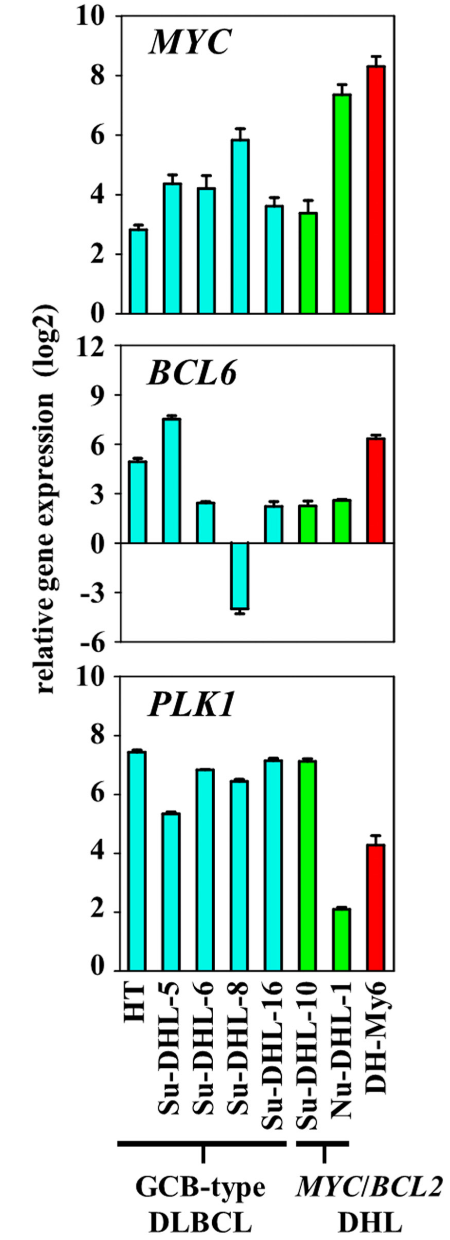

Supplementary Figure 1: Gene expression analyses of *MYC*, *BCL6*, and *PLK1*. Relative mRNA expression levels in DH-My6 and seven DLBCL cell lines with a GCB phenotype, including two cell lines with *MYC* and *BCL2* rearrangements, were calculated using the $2^{-\Delta Ct}$ method with the *ACTB* gene used as a housekeeping control. Data are shown as the mean ± SEM of three independent experiments.

A

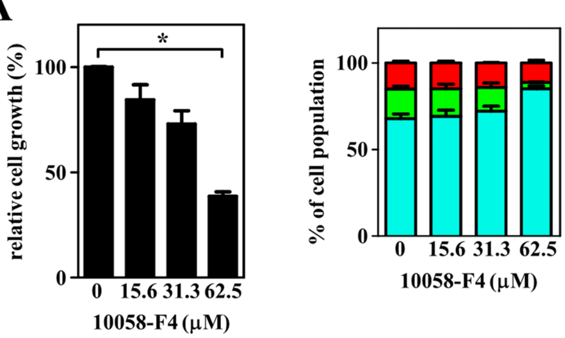

10058-F4 (μM)	% of cell population 0	15.6	31.3	62.5
G0/G1	67.77	69.05	72.10	85.08**
S	17.11	16.06	13.67	3.67**
G2/M	15.12	14.89	14.23	11.25**

B

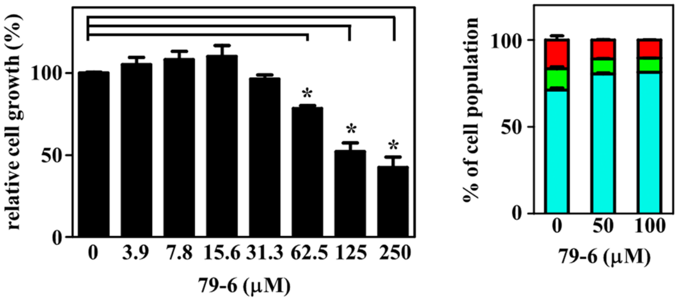

79-6 (μM)	% of cell population 0	50	100
G0/G1	71.09	80.42**	81.37**
S	12.28	8.64*	8.25*
G2/M	16.63	10.94	10.38

Supplementary Figure 2: Effects of the MYC inhibitor 10058-F4 and BCL6 inhibitor 79-6 on cell growth and the cell cycle in DH-My6 cells. Cells were treated with 10058-F4 **(A)** or 79-6 **(B)** for 48 h at the indicated concentrations, and cell growth assays (left panels) and cell cycle analyses (middle panels) were performed. The numbers of viable cells are normalized as a percentage of the viable cell numbers of DMSO-treated controls. Percentages of the cell population in each stage of the cell cycle are presented outside the graph (right panels). All experiment were repeated independently three times and data are expressed as the mean ± SEM. Significant expression differences are shown as *P < 0.05; **P <0.01.

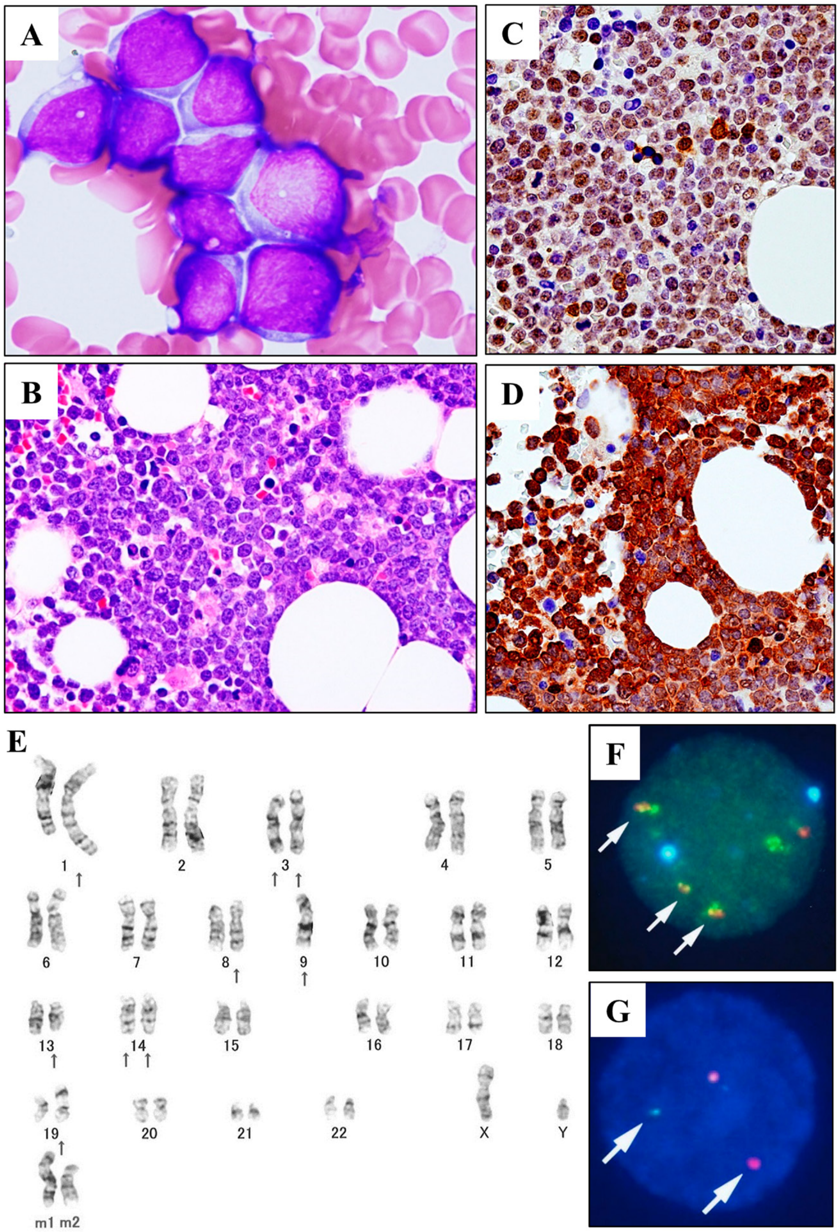

Supplementary Figure 3: Pathology findings in primary lymphoma cells. **(A)** Smear of the bone marrow aspirate, showing infiltration of medium-to-large-sized lymphoma cells (May-Giemsa staining). **(B)** Specimen of the bone marrow aspirate clot (Hematoxylin and eosin stain). **(C)** Anti-MYC immunostaining, showing that the lymphoma cells were positive for MYC. **(D)** Anti-BCL6 immunostaining, showing that the lymphoma cells were also positive for BCL6. **(E)** G-banding karyotype, showing 47, XY, ins(1;?)(q21:?), add(3)(p25), der(3)t(3;14)(q27;q32), add(8)(q24), -9, add(9)(p11), del(13)(q?), der(14)t(8;14)(q24;q32), add(19)(p13), +mar1, +mar2. **(F)** FISH analysis with an *IGH–MYC* dual-color probe, showing fusion signals for the *IGH–MYC* rearrangement (arrows). The green signal corresponds to the normal *IGH* allele, red to the normal *MYC* allele, yellow to the *IGH–MYC* fusion gene, and blue signal to the centromeric region of chromosome 8. **(G)** FISH analysis for *BCL6* gene rearrangement with the break-apart probe, showing split signals for *BCL6* (arrows). Yellow signal corresponds to the intact, nonrearranged *BCL6* locus, while separate red (5' *BCL6* FISH DNA probe) and green (3' *BCL6* FISH DNA probe) signals indicate the *BCL6* rearrangement.

Supplementary Table 1: Sequences of primers used for LD-PCR and RT-qPCR

Target region	Primer name	Sequence (5'→3')	Nucleotide position	Accession number
**LD-PCR**				
MYC exon 2	MYC/M6	ACAGTCCTGGATGATGATGTTTTTGATGAAGGTCT	8228–8194	NG_007161.2
IGH Sα	αR2	TGGTTTCTGAACATGCTCCTTAGATAGG	105708828–105708855	NC_000014.9
BCL6 exon 1	BCL6/09	TTCGCCAGGGTTCCAATAACACGGCATCATAAAGG	187743613–187743647	NC_000003.12
IGH Sμ	JXI	CCCATGCCTTCCAAAGCGATT	105860988–105860966	NC_000014.9
**RT-qPCR**				
MYC	MYC-F	AGCGACTCTGAGGAGGAACAAG	1953–2082	NM_002467. 5
	MYC-R	TGTGAGGAGGTTTGCTGTGG		
BCL6	BCL6-F	CGGTGAGAAACCCTATCGTTG	2072–2126	NM_001706.4
	BCL6-R	CGAGTGTGGGTTTTCAGGTTG		
PLK1	PLK1-F	CCCATCTTCTGGGTCAGCAAG	1380–1468	NM_005030. 5
	PLK1-R	AAGAGCACCCCCACGCTGTT		
β-actin	ACTB-F	GTGGATCAGCAAGCAGGAGTATGA	1257–1342	NM_001101. 4
	ACTB-R	TAGGTTTTGTCAAGAAAGGGTGTAA		

Abbreviations: LD–PCR, long-distance polymerase chain reaction; RT–qPCR, quantitative reverse-transcription PCR.
